# Cross-sectional and longitudinal associations of serum Cysteine-rich 61 with severity and prognosis among community-acquired pneumonia patients in China

**DOI:** 10.3389/fmed.2022.939002

**Published:** 2022-08-11

**Authors:** Meng-Xing Yao, Jia-Yi Cheng, Ying Liu, Jing Sun, Dong-Xu Hua, Qi-Yuan He, Hong-Yan Liu, Lin Fu, Hui Zhao

**Affiliations:** ^1^Department of Respiratory and Critical Care Medicine, Second Affiliated Hospital of Anhui Medical University, Hefei, China; ^2^Department of Toxicology, Anhui Medical University, Hefei, China

**Keywords:** Cyr61, community-acquired pneumonia, severity, prognosis, inflammation, cohort study

## Abstract

**Background:**

Cysteine-rich 61 (CYR61) is implicated in many pulmonary diseases. However, the relationship between CYR61 and community-acquired pneumonia (CAP) patients was unknown. This research aimed to estimate the correlations of serum CYR61 with severity and prognosis in CAP patients through a prospective cohort study.

**Methods:**

All 541 CAP patients were enrolled in this study. Fasting venous blood was collected. Clinical characteristics and demographic information were obtained. CYR61 and inflammatory cytokines were detected in serum using ELISA.

**Results:**

Serum CYR61 was gradually increased in parallel with severity scores in CAP patients. Correlative analysis indicated that serum CYR61 was strongly associated with many clinical parameters in CAP patients. Moreover, mixed logistic and linear regression models found that there were positive correlations between serum CYR61 and CAP severity scores after adjusted for age, BMI, and respiratory rate. Stratified analyses suggested that age affected the associations between serum CYR61 and severity scores. On admission, higher serum CYR61 levels elevated the risks of mechanical ventilation, vasoactive agent, ICU admission, death, and longer hospital stays during hospitalization. Moreover, serum CYR61 in combination with severity scores upregulated the predictive capacities for severity and death than single serum CYR61 or severity scores in CAP patients.

**Conclusion:**

There are significantly positive dose-response associations of serum CYR61 on admission with the severity and adverse prognostic outcomes, demonstrating that CYR61 is involved in the pathophysiology of CAP. Serum CYR61 may be used as a potential biomarker for the diagnosis and prognosis in CAP patients.

## Introduction

Community-acquired pneumonia (CAP), one of the most frequent infectious diseases, is a significant cause of mortality worldwide (1, 2). At the present, CAP is still one of the most significant reasons for mortality in children under the age of 5 years and the elderly, accounting for more than 2 million deaths globally annually (3–5). Simultaneously, CAP is a principal consideration for hospitalization annually (6). It is generally believed that the bacterium that triggers CAP is *Streptococcus pneumoniae*. Moreover, pathogen detection reveals that exposure to atypical pathogens, such as *Legionella pneumophila, Staphylococcus aureus*, gram-negative bacilli, fungal, or parasitic organisms, can also evoke CAP (7–9). Despite the quick progress of therapeutic methods and detected technology, the mortality of CAP is still high in the whole world (10, 11). Therefore, earlier CAP diagnosis can effectively improve targeted therapy and facilitate the rational use of clinical drugs. In addition, it helps reduce treatment costs and adverse side effects for CAP patients.

Cysteine-rich protein 61 (CYR61), also known as CCN1, is a member of the CCN family proteins. CYR61 is a secretory protein, which is related to the extracellular matrix (12). Recent studies have found that CYR61 exerts significant roles in maintaining cell survival, proliferation, adhesion, migration, angiogenesis, apoptosis, aging, and senescence and in activating many singling transduction pathways (13–15). In addition, the associations between CYR61 and pulmonary diseases are getting more attention. A previous study has revealed that CYR61 is richly expressed in pulmonary epithelial cells (16). Several reports indicated that CYR61 is increased in different factors-evoked acute lung injury models (17, 18). On the contrary, CYR61 is elevated in lung tissues of idiopathic pulmonary fibrosis patients (19). Moreover, two recent reports suggested that the expressions of mRNA and protein are upregulated in the lung tissues of chronic obstructive pulmonary disease (COPD) patients compared with non-smokers (20, 21). However, the association between CYR61 and CAP was unknown.

So far, there was no relative report about the relationship between CYR61 and CAP. Nevertheless, the expression of CYR61 is elevated in patients with coronavirus disease 2019 (22). In addition, many studies have demonstrated that the levels of CYR61 are upregulated in inflammatory diseases, such as Graves’ orbitopathy, rheumatoid arthritis, and pulmonary hypertension (23–25). CAP is also one of the inflammatory and infectious diseases. So, we conjectured that CYR61 may involve in the pathophysiology of CAP. Moreover, the relationships between serum CYR61 and the prognostic outcomes were obscure in CAP patients. Therefore, the purpose of this study was to evaluate the relationships of serum CYR61 with the clinical physiological indices and the different prognostic outcomes among CAP patients based on a prospective cohort study. Our results provided evidence that serum CYR61 on admission is positively associated with the severity and poor prognosis in CAP patients.

## Materials and methods

### Study design and data collection

In total, 541 patients diagnosed with CAP were enrolled in this research. This research was performed at the Department of Respiratory and Critical Care Medicine, the Second Affiliated Hospital of Anhui Medical University, Hefei city, Anhui province, China. From September 2020 to July 2021, 541 available serum specimens were collected from CAP patients. The inclusion criteria were as follows: (1) newly diagnosed CAP patients who met CAP diagnostic criteria (26, 27); (2) all subjects who participated in this research and completed the follow-up research on their own will; and (3) all CAP patients who had not undergone any treatment and intervention before they participate in this research. The exclusion criteria were as follows: (1) less than 18 years old; (2) pregnant women; (3) other drugs, such as antibiotics, antiviral drugs, and glucocorticoids, administered over the week; (4) CAP patients complicated with other diseases, such as autoimmune disease, pulmonary malignancy, COPD, several respiratory infectious diseases, asthma, and bronchiectasis; (5) hospital stay less than 1 week; and (6) patients who had an attack or suffered from hospitalization in the last 6 months. For studying serum biomarkers, fasting blood specimens from CAP patients were collected before intervention or treatment (28, 29). Moreover, demographic information and clinical characteristics were obtained from the electronic medical record system in the hospital (30, 31). The severity of CAP was evaluated through CAP severity scores (CURB-65, CRB-65, SMART-COP, PSI, CURXO, and APACHE II) (32, 33).

### Enzyme-linked immunosorbent assay

Before the collection of peripheral blood samples, all CAP patients must fast and limit water intake. Then, the peripheral blood specimens were centrifuged at 3,500 RPM at 4°C. The serum was packed and preserved at −-80°C in a super cold refrigerator spare (34, 35). CYR61 commercial ELISA kits (CSB-E13884h) were purchased from Cusabio, Wuhan, China.^1^ Tumor necrosis factor α (TNF-α; JYM0110Hu) ELISA kits were obtained from Wuhan ColorfulGene Biological Technology Co., Ltd.^2^ The concentrations of CYR61 and inflammatory cytokines were detected in serum through ELISA based on the previous studies with minor adjustments (36–38).

### Statistical analysis

All statistical analyses were performed through the SPSS 13.0 software. The mean (standard error) was used to describe the continuous variables of the normal distribution. The median was used to show the continuous variables of the non-normal distribution. The categorical variables were expressed using frequency or percentage. The continuous variables were compared with Student’s *t*-test and the one-way ANOVA test. Additionally, the categorical variables were compared using the chi-square (χ^2^) test. The relationships between serum CYR61 and the markers of pathophysiological characteristics were analyzed in accordance with the Spearman’s correlation coefficient or Pearson’s rank correlation. Besides, binary logistical regression analysis and multinomial logistical regression analysis were used to analyze the relationships between serum CYR61 and CAP severity scores with or without adjustment for age. Moreover, the associations between serum CYR61 and the prognostic outcomes were estimated through the chi-square test and mixed logistical regression models. To exclude potential confounding factors on the relationships between serum CYR61 and CAP severity scores, stratified analyses were conducted in mixed logistic regression models. Effect modification by each covariate in relationships with serum CYR61 and CAP severity scores was estimated through adopting an interaction term of serum CYR61 multiplied by the covariates in the logistic regression mixed models. A *P*-value less than 0.05 (two-tailed) or 95% confidence interval (CI) not including 1 (for ordinal regression) was considered to indicate statistical significance.

## Results

### Characteristics of study population

Demographics information and clinical characteristics were compared and analyzed. As shown in Table 1, the median of age was 62.0 years, and female subjects accounted for 41.2% among all CAP subjects. There were 108 (20.0%) smokers in CAP patients. The average body mass index (BMI) was 22.3. The means of heart rate, respiratory rate, and saturation of peripheral oxygen were 89.4 beats per min, 19.8 breaths per min, and 94.8%, respectively. The mean of body temperature was 36.7°C on admission. The average systolic pressure and diastolic pressure was 125.2 and 75.2 mmHg, respectively. Then, the comorbidities were assessed. Among the 541 CAP patients, 146 patients presented with hypertension, 59 with diabetes mellitus, 50 with cerebral infarction, 28 with coronary heart disease, and 6 with bronchitis (Table 1). Moreover, routine blood indexes such as white blood cell (WBC), neutrophil, lymphocyte, eosinophil, basophil, procalcitonin, D-dimer, C-reactive protein (CRP), interleukin-6 (IL-6), and TNF-α were measured in CAP patients. In addition, the indicators of liver function, renal function, and myocardial function were detected. Finally, CAP severity scores were estimated in CAP patients. As shown in Table 1, the number of mild CAP cases was 428 (79.1%), and the number of severe CAP subjects was 113 (20.9%). The means of CURB-65, CRB-65, PSI, SMART-COP, and APACHE II were 1.0, 1.0, 62.0, 1.0, and 6.0 scores, respectively (Table 1). Moreover, we found that age and inflammatory cytokines, as well as CAP severity scores, were gradually elevated with increasing serum CYR61 in CAP patients.

**TABLE 1:** Demographic characteristics of participators at baseline.

Characteristic	All participators	Tertile of serum CYR61			*P*
		Tertile 1 (<111.4 ng/mL)	Tertile 2 (111.4∼151.8 ng/mL)	Tertile 3 (>151.8 ng/mL)	
*N*	541	180	181	180	
Age, years	62.0 (48.0, 73.0)	56.0 (42.0, 68.0)	63.0 (47.0, 74.0)	65.0 (55.0, 76.0)	< 0.021
Female, *n* (%)	223 (41.2)	71 (39.3)	87 (48.3)	65 (36.0)	0.313
Body mass index	22.3 ± 0.29	22.7 ± 0.43	22.8 ± 0.55	21.2 ± 0.50	**0.048**
Smoker, *n* (%)	108 (20.0)	36 (20.2)	33 (18.0)	39 (21.6)	0.801
Heart rate (beats per min)	89.4 ± 1.11	89.6 ± 1.87	89.5 ± 1.85	89.2 ± 2.04	0.983
Respiratory rate (breaths per min)	19.8 ± 0.21	19.7 ± 0.34	19.2 ± 0.0.18	20.6 ± 0.49	**0.019**
Oxygen saturation (%)	94.8 ± 0.55	96.2 ± 0.41	95.1 ± 0.53	93.0 ± 1.50	0.052
Temperature (°C)	36.7 ± 0.04	36.8 ± 0.08	36.7 ± 0.07	36.6 ± 0.06	0.228
Systolic pressure (mmHg)	125.2 ± 0.72	123.0 ± 1.84	125.3 ± 2.22	127.1 ± 2.08	0.361
Diastolic pressure (mmHg)	75.2 ± 0.72	74.3 ± 1.08	74.7 ± 1.29	76.6 ± 1.35	0.365
Hypertension, *n* (%)	146 (27.0)	44 (24.7)	53 (29.2)	49 (27.0)	0.733
Diabetes mellitus, *n* (%)	59 (10.9)	14 (7.9)	23 (12.7)	22 (12.4)	0.328
Cerebral infarction, *n* (%)	50 (9.2)	12 (6.7)	16 (9.0)	22 (12.4)	0.249
Coronary heart disease, *n* (%)	28 (5.2)	10 (5.6)	10 (5.5)	8 (4.4)	0.875
Bronchitis, *n* (%)	6 (1.1)	6 (3.4)	0	0	0.003
White blood cell (10^9^/L)	8.3 ± 0.25	8.2 ± 0.42	8.2 ± 0.41	8.4 ± 0.47	0.955
Neutrophil (10^9^/L)	6.3 ± 0.24	6.2 ± 0.40	6.1 ± 0.39	6.6 ± 0.46	0.997
Lymphocyte (10^9^/L)	1.3 ± 0.05	1.3 ± 0.07	1.4 ± 0.11	1.0 ± 0.07	0.188
Monocyte (10^9^/L)	0.6 ± 0.03	0.6 ± 0.04	0.6 ± 0.05	0.6 ± 0.06	0.895
Eosinophil (10^9^/L)	0.05 (0.01, 0.11)	0.04 (0, 0.11)	0.07 (0.02, 0.14)	0.04 (0, 0.11)	0.967
Basophil (10^9^/L)	0.02 (0.01, 0.03)	0.02 (0.01, 0.03)	0.02 (0.01, 0.03)	0.02 (0.01, 0.03)	0.449
Procalcitonin (ng/L)	0.08 (0.03, 0.50)	0.08 (0.03, 0.49)	0.07 (0.03, 0.20)	0.10 (0.03, 0.65)	0.116
D-dimer (mg/L)	0.82 (0.41, 2.29)	0.66 (0.35, 1.34)	0.71 (0.42, 2.34)	1.29 (0.61, 2.94)	0.006
C-reactive protein (mg/L)	59.7 (7.7, 142.7)	65.6 (15.3, 159.9)	50.0 (6.9, 138.2)	36.0 (6.3, 143.8)	0.865
Interleukin-6 (pg/mL)	14.8 (4.1, 42.9)	9.1 (2.5, 22.0)	15.2 (4.6, 41.1)	23.5 (4.6, 67.0)	0.367
Tumor necrosis factor α (pg/mL)	7.5 (4.5, 22.6)	5.7 (4.3, 21.0)	6.1 (4.3, 28.7)	10.1 (5.0, 36.1)	0.025
CURB-65	1.0 (0, 2.0)	0.6 ± 0.09	1.0 ± 0.10	1.6 ± 0.15	< 0.001
CRB-65	1.0 (0, 2.0)	0.5 ± 0.07	0.8 ± 0.09	1.3 ± 0.12	< 0.001
PSI	62.0 (40.0, 87.0)	48.8 ± 3.23	67.2 ± 4.34	81.4 ± 4.77	< 0.001
CURXO [Mild, *n* (%)]	428 (79.1)	167 (92.8)	148 (81.8)	113 (62.9)	0.049
SMART-COP	1.0 (0, 2.0)	1.1 ± 0.15	1.6 ± 0.22	2.3 ± 0.24	< 0.001
APACHE II	6.0 (4.0, 9.0)	5.8 ± 0.41	6.8 ± 0.71	10.0 ± 0.81	< 0.001

Bold values indicate statistical significance.

### The levels of serum Cysteine-rich 61 in community-acquired pneumonia patients with different severity scores

**FIGURE 1:**
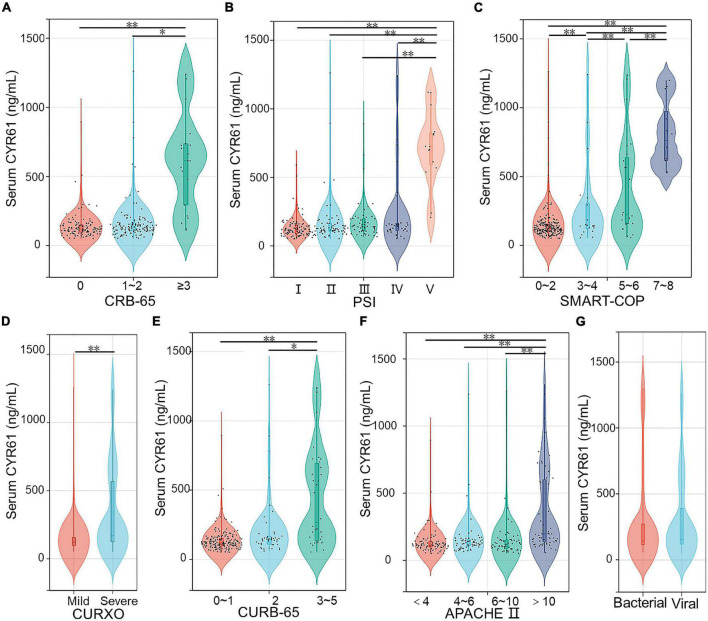
The levels of serum CYR61 in CAP patients. **(A–G)** The level of serum CYR61 was measured using ELISA. **(A)** The levels of serum CYR61 in CAP patients with different CRB-65 score. **(B)** The levels of serum CYR61 in CAP patients with different PSI score. **(C)** The levels of serum CYR61 in CAP patients with different SMART-COP score. **(D)** The levels of serum CYR61 in CAP patients with different CURXO score. **(E)** The levels of serum CYR61 in CAP patients with different CURB-65 score. **(F)** The levels of serum CYR61 in CAP patients with different APACHE II score. **(G)** The levels of serum CYR61 in CAP patients with bacterial infection or viral infection. *P < 0.05, ^**^P < 0.01.

The levels of serum CYR61 were detected in CAP patients with different severity scores. As shown in Figure 1A, CAP patients were divided into three grades in accordance with the CRB-65 score. The results suggested that the level of serum CYR61 was higher in the grade of ≥ 3 scores than those in the grades of 0 score and 1–2 scores in CAP patients. According to the PSI score, the content of serum CYR61 was highest in CAP patients with highest PSI score (Figure 1B). Moreover, as shown in Figure 1C, the concentration of serum CYR61 was highest in CAP patients with 7–8 scores of SMART-COP. The level of serum CYR61 was gradually increased in parallel with SMART-COP score in CAP patients. In addition, CAP patients were separated into mild and severe cases on the basis of CURXO score. As shown in Figure 1D, serum CYR61 was significantly increased in severe patients compared with mild cases. According to the CURB-65 score, the content of serum CYR61 was elevated in the grade of 3–5 scores compared with the grades of 0–1 score and 2 score among CAP patients (Figure 1E). In addition, the levels of CYR61 were compared in CAP patients with different APACHE II scores. We found that the level of serum CYR61 was higher in the grade of more than 9 scores than those in other grades (Figure 1F). Finally, the level of serum CYR61 was compared in CAP patients between bacterial infection and viral infection. The results indicated that there was no obvious difference of serum CYR61 in CAP patients between bacterial infection and viral infection (Figure 1G).

### Correlations between serum Cysteine-rich 61 and the clinical characteristics in community-acquired pneumonia patients

**FIGURE 2:**
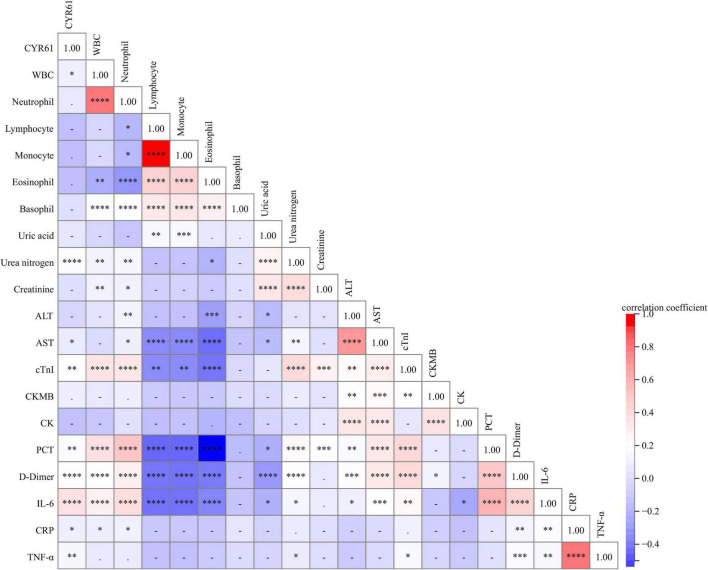
The relationships between serum CYR61 and clinical characteristics in CAP patients. The relationships between serum CYR61 and clinical characteristics were analyzed in CAP patients through Spearman’s or Pearson’s correlative analysis. Different colors indicate different correlation coefficients. Red color indicates the positive correlation, and blue color indicates the negative correlation. The darker the color, the stronger the correlation. * *P* < 0.05, ** *P* < 0.01, *** *P* < 0.001, **** *P* < 0.0001.

The associations of serum CYR61 and the indices of clinical characteristics were analyzed through Spearman’s correlation coefficient or Pearson’s rank correlation in CAP patients. As shown in Figure 2, although there was no obvious association of serum CYR61 with neutrophil, lymphocyte, monocyte, eosinophil, and basophil, serum CYR61 was weakly and positively associated with WBC (*r* = 0.148; *P* = 0.044) in CAP patients. Moreover, the associations of serum CYR61 with the indices of liver function, renal function, and myocardial function were assessed. The results indicated that serum CYR61 was positively related with urea nitrogen (*r* = 0.256; *P* = 0.003), aspartate aminotransferase (AST; *r* = 0.197; *P* = 0.033), and cardiac troponin I (cTnI; *r* = 0.221; *P* = 0.008) in CAP patients (Figure 2). We also found that serum CYR61 was positively correlated with procalcitonin (PCT; *r* = 0.414; *P* = 0.015) and D-dimer (*r* = 0.490; *P* < 0.001) in CAP patients. Ultimately, the correlations between serum CYR61 and inflammatory cytokines were evaluated. As shown in Figure 2, serum CYR61 was positively correlated with IL-6 (*r* = 0.356; *P* < 0.001), CRP (*r* = 0.260; *P* = 0.011), and TNF-α (*r* = 0.302; *P* = 0.007) in CAP patients.

### Associations between Cysteine-rich 61 and severity scores in community-acquired pneumonia patients

The associations between serum CYR61 and CAP severity scores were assessed through mixed linear and logistic regression models in CAP patients. In the mixed linear regression model, age, BMI, and respiratory rate were adjusted. The results indicated that each 1 ng/ml increase of serum CYR61 was correlated with 1.005 score (95%CI: 1.001–1.010), 1.006 score (95%CI: 1.001–1.011), 1.006 score (95%CI: 1.000–1.010), 1.006 score (95%CI: 1.002–1.010), and 1.133 score (95%CI: 1.001–1.010) changes for CURB-65, CRB-65, CURXO, SMART-COP, and APACHE II, respectively (Table 2). Moreover, mixed logistic regression model found that the severity scores of PSI and APACHE II were gradually increased in line with the tertiles of serum CYR61. Compared with the subjects from the lowest serum CYR61 group, the cases from the highest serum CYR61 group had 3.303 times increase of CURXO in CAP patients (Table 2).

**TABLE 2:** Associations between serum CYR61 and CAP severity scores.

Variables	Estimated changes continues serum CYR61	Estimated changes (95% CI) by tertiles of serum CYR61			*P* trend
		Tertile 1	Tertile 2	Tertile 3	
*N*	541	181	181	180	
CURB-65	**1.005 (1.001, 1.010)**	0 (Ref)	1.917 (0.613, 5.996)	1.794 (0.564, 5.707)	0.098
CRB-65	**1.006 (1.001, 1.011)**	0 (Ref)	2.068 (0.458, 9.340)	2.963 (0.701, 12.519)	0.082
PSI	1.007 (0.999, 1.014)	0 (Ref)	**1.993 (1.014, 4.762)**	**2.069 (1.114, 5.257)**	**0.011**
CURXO (Severe)	**1.006 (1.002, 1.011)**	0 (Ref)	4.872 (0.956, 24.827)	**3.303 (1.596, 18.320)**	**0.021**
SMART-COP	**1.006 (1.002, 1.010)**	0 (Ref)	1.266 (0.526, 3.047)	0.856 (0.333, 2.200)	0.123
APACHE II	**1.133 (1.001, 1.012)**	0 (Ref)	**2.958 (1.190, 7.352)**	**4.525 (2.626, 13.719)**	**0.001**

Models were adjusted for age, BMI and respiratory rate. Bold values indicate statistical significance.

In view of the potential confounding factors, the influences of age, BMI, and respiratory rate on CAP severity scores were further estimated through subgroup analysis. Stratified analysis suggested that age modified the relationships between serum CYR61 and CURXO score (*P* interaction <0.05; Table 3). In CAP patients aged less than 62.0 years, serum CYR61 was positively associated with CURXO (OR = 1.008; 95%CI: 1.001–1.014). Moreover, serum CYR61 was positively related with CURXO (OR = 1.113; 95%CI: 1.005–1.345) in CAP patients more than 62.0 years. Furthermore, age also affected the associations between serum CYR61 and APACHE II score (*P* interaction <0.01) among CAP patients (Table 3). Moreover, there was no obvious effect of other potential variables on the associations between serum CYR61 and CAP severity scores.

**TABLE 3:** Stratified analysis for the associations between serum CYR61 and severity scores.

Stratification characteristic		PSI	CURB-65	CRB-65	CURXO	SMART-COP	APACHE II
Age							
	≤62.0 years	**1.216 (1.021, 2.387)**	0.956 (0.963, 1.036)	**1.001 (0.985, 1.018)** *	**1.008 (1.001, 1.014)** *	1.000 (0.991, 1.008)	0.996 (0.985, 1.007)
	>62.0 years	**1.261 (1.046, 2.484)**	**1.013 (1.000, 1.025)** **	**1.008 (1.001, 1.015)** *	**1.113 (1.005, 1.345)** *	1.004 (0.999, 1.008)	1.002 (0.997, 1.007)
	*P*_*interaction*_	0.727	0.099	0.170	**0.025**	0.283	**0.003**
BMI							
	≤22.2	1.004 (0.999, 1.009)	**1.014 (1.001, 1.058)** *	**1.006 (1.001, 1.011)** *	1.026 (1.001, 1.114)*	1.003 (1.000, 1.007)	**1.115 (1.001, 1.216)** *
	>22.2	1.009 (0.999, 1.019)	1.010 (0.998, 1.022)	1.006 (1.001, 1.011)	1.118 (1.011, 1.217)*	1.004 (0.998, 1.010)	**1.214 (1.007, 1.535)** *
	*P*_*interaction*_	0.065	0.112	0.387	0.225	0.114	0.094
Respiratory rate							
	≤20.0	**1.003 (1.001, 1.015)** *	**1.125 (1.003, 1.354)** ***	**1.004 (1.002, 1.016)** ***	**1.114 (1.002, 1.254)** **	1.003 (0.998, 1.124)	**1.005 (1.003, 1.017)** ***
	>20.0	1.049 (0.996, 1.104)	1.055 (0.995, 1.120)	1.038 (0.984, 1.096)	**1.112 (1.008, 1.354)** *	1.012 (0.089, 1.158)	**1.100 (1.007, 1.302)** **
	*P*_*interaction*_	0.087	0.154	0.118	0.153	0.113	0.125

Models were adjusted for age, BMI, and respiratory rate.

*P < 0.05, **P < 0.01, ***P < 0.001. Bold values indicate statistical significance.

### Associations of serum Cysteine-rich 61 with the prognostic outcomes in community-acquired pneumonia patients

During hospitalization, prognostic outcomes, such as mechanical ventilation, vasoactive agent usage, ICU admission, death, and longer hospital stays, were observed and tracked up in CAP patients. Among the 541 CAP patients, there was 14 patients with mechanical ventilation in tertile 1 group, 20 patients in tertile 2 group, and 51 patients in tertile 3 group. Logistic regression analysis indicated that the relative risk (RR) in tertile 3 group (RR = 4.649; 95% CI: 1.890–11.435) was obviously higher than that in tertile 1 group. Moreover, age was adjusted. The adjusted RR in tertile 3 group (RR = 3.556; 95% CI: 1.389–9.103) was increased compared with tertile 1 group (Table 4). In addition, 6 (3.3%) cases were with vasoactive agent usage in tertile 1 group, 10 (5.5%) cases were in tertile 2 group, and 32 cases were in tertile 3 group. The RR for vasoactive agent usage was 6.283 (95% CI: 1.761–22.417) in tertile 3 group. The adjusted RR for vasoactive agent usage was 4.911 (95% CI: 1.338–18.021) in tertile 3 group (Table 4). Besides, the RR of ICU admission (4.179; 95% CI: 1.772–9.856) and adjusted RR (3.195; 95% CI: 1.300–7.855) were higher in tertile 3 group compared with tertile 1 group. Additionally, the cases of death were 4 (2.2%) in tertile 1 group, 8 (4.4%) in tertile 2 group, and 28 (15.6%) in tertile 3 group. The RR and adjusted RR were obviously elevated in higher CYR61 group than those in lower CYR61 group (Table 4). Finally, the length of hospital stay was divided into two grades, namely, longer hospital stays, ≥ 75% quantile; and lower hospital stays, <75% quantile. Logistic regression analysis found that the cases and RR were increased in tertile 3 group compared with tertile 1 group (Table 4).

**TABLE 4:** Adjusted relative risk for prognostic outcomes by tertiles of serum CYR61.

Variables	Serum CYR61			*P* trend
	Tertile 1	Tertile 2	Tertile 3	
*N*	180	181	180	
Mechanical ventilation				
*N*, (%)	14 (7.8%)	20 (11.0%)	51 (28.3%)	< 0.001
Unadjusted RR (95% CI)	Ref. (1)	1.483 (0.538, 4.088)	4.649 (1.890, 11.435)	
Adjusted RR (95% CI)	Ref. (1)	1.324 (0.460, 3.813)	3.556 (1.389, 9.103)	
Vasoactive agent				
*N*, (%)	6 (3.3%)	10 (5.5%)	32 (17.8%)	0.001
Unadjusted RR (95% CI)	Ref. (1)	1.706 (0.395, 7.367)	6.283 (1.761, 22.417)	
Adjusted RR (95% CI)	Ref (1)	1.631 (0.367, 7.240)	4.911 (1.338, 18.021)	
ICU admission				
*N*, (%)	18 (10.0%)	20 (11.0%)	53 (29.4%)	< 0.001
Unadjusted RR (95% CI)	Ref. (1)	1.282 (0.481, 3.415)	4.179 (1.772, 9.856)	
Adjusted RR (95% CI)	Ref. (1)	1.151 (0.413, 3.205)	3.195 (1.300, 7.855)	
Death				
*N*, (%)	4 (2.2%)	8 (4.4%)	28 (15.6%)	0.001
Unadjusted RR (95% CI)	Ref. (1)	2.071 (0.370, 11.610)	8.120 (1.788, 36.884)	
Adjusted RR (95% CI)	Ref. (1)	1.931 (0.335, 11.144)	6.163 (1.320, 28.784)	
Longer hospital stays				
*N*, (%)	20 (11.1%)	28 (15.5%)	63 (35.0%)	< 0.001
Unadjusted RR (95% CI)	Ref. (1)	1.475 (0.617, 3.523)	4.222 (1.918, 9.297)	
Adjusted RR (95% CI)	Ref. (1)	1.369 (0.559, 3.352)	3.485 (1.546, 7.852)	

RR: Relative risk.

The length of hospital stay was divided into two groups: longer hospital stays, ≥14 days; lower hospital stays, <14 days.

### The predictive power of serum Cysteine-rich 61 for severity and death in community-acquired pneumonia patients

**FIGURE 3:**
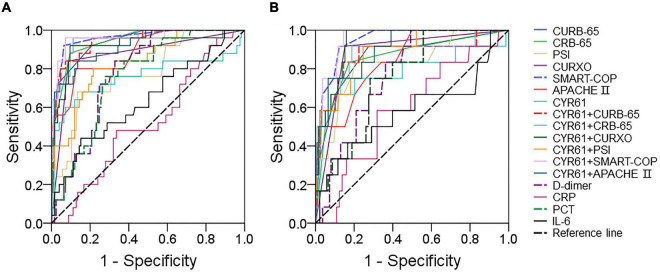
The predictive capacities of different biomarkers for severity and death. **(A,B)** The predictive powers for severity and death were evaluated through ROC curve in many biomarkers. **(A)** The predictive power for severity was analyzed through ROC curve among CAP severity scores, serum CYR61, markers of inflammation, and infection. **(B)** The predictive power for death was estimated *via* ROC curve among CAP severity scores, serum CYR61, markers of inflammation, and infection.

The predictive capacity of serum CYR61 for severity was evaluated in CAP patients through receiver operating characteristic (ROC) area under the curve (AUC). As shown in Figure 3A, the predictive powers of CYR61, CAP severity scores, and serum CYR61 in combination with CAP severity scores for severe patients were as follows: CYR61, 0.811; CURB-65, 0.926; CURB-65 + CYR61, 0.933; CRB-65, 0.928; CRB-65 + CYR61, 0.930; PSI, 0.790; PSI + CYR61, 0.827; CURXO, 0.884; D-dimer, 0.780; PCT, 0.759; IL-6, 0.637; CRP, 0.518; CURXO + CYR61, 0.920; SMART-COP, 0.969; SMART-COP + CYR61, 0.970; APACHE II, 0.907; and APACHE II + CYR61, 0.933. The optimal cutoff concentration of serum CYR61 for severe patients was 182.72 ng/ml. The specificity was 59.5%, and the sensitivity was 85.3%. Moreover, the predictive powers of CYR61, CAP severity scores, and serum CYR61 in combination with CAP severity scores for death were evaluated in CAP patients. The results were as follows: CURB-65, 0.908; CURB-65 + CYR61, 0.913; CRB-65, 0.891; CRB-65 + CYR61, 0.902; PSI, 0.872; CYR61, 0.786; D-dimer, 0.772; PCT, 0.754; CRP, 0.589; IL-6, 0.579; PSI + CYR61, 0.910; CURXO, 0.902; CURXO + CYR61, 0.946; SMART-COP, 0.946; SMART-COP + CYR61, 0.960; APACHE II, 0.895; and APACHE II + CYR61, 0.948 (Figure 3B). The optimal cutoff content of serum CYR61 for death was 185.75 ng/ml. The specificity was 70.0%, and the sensitivity was 82.1%.

## Discussion

This research mainly estimated the correlations of serum CYR61 on admission with the severity scores and poor prognostic outcomes among CAP patients. The specific findings include serum CYR61 on admission was gradually increased in parallel with CAP severity scores; serum CYR61 on admission was positively correlated with the severity scores among CAP patients; and higher serum CYR61 on admission upregulated the risks of adverse prognostic outcomes among CAP patients. The predicative capacities of serum CYR61 in combination with PSI, CURXO, and APACHE II were higher compared with single serum CYR61 or severity scores among CAP patients.

CYR61 is a secretory protein, which is associated with extracellular matrix (12). Compelling evidence has revealed that CYR61 plays an important role in maintaining the physiological functions of cells normally (13–15). Increasing studies have found that CYR61 is highly expressed in pulmonary epithelial cells and is involved in many pulmonary diseases (16). Previous studies have demonstrated that the expression of pulmonary CYR61 is increased in many inflammatory diseases, such as acute lung injury, idiopathic pulmonary fibrosis, pulmonary hypertension, and COPD (17–21, 23). Given CAP is one of inflammatory and infectious diseases, we conjecture that CYR61 may be implicated in the pathophysiology progression of CAP. Then, the level of serum CYR61 was measured. We found that the level of serum CYR61 was increased in CAP patients compared with control subjects. In addition, the level of serum CYR61 was gradually elevated in parallel with CAP severity scores. Moreover, correlative analysis indicated that the level of serum CYR61 was strongly correlated with many clinical parameters in CAP patients. Moreover, mixed linear and logistic regression models confirmed that serum CYR61 was positively associated with CAP severity scores. Besides, we found that serum CYR61 was positively correlated with indicators of renal function, liver function, and inflammation among CAP patients. Interestingly, although there was no association of serum CYR61 with neutrophil, lymphocyte, monocyte, eosinophil, and basophil, serum CYR61 was weakly and positively correlated with the number of WBC in CAP patients. Our previous research has found that the number of lymphocyte was decreased, and the count of neutrophil was increased in CAP patients. The total WBC was slightly elevated in CAP patients compared with healthy volunteers (39). CAP led to contrary changes of lymphocyte and neutrophil. Maybe, this was the reason for the weak correlation between serum CYR61 and WBC. Therefore, these results hinted that serum CYR61 on admission is positively associated with CAP severity scores.

More and more studies have found that there are associations between CYR61 and many prognostic outcomes in a series of diseases. CYR61 is involved in the transduction of growth factor and hormone signaling, and the levels of CYR61 are always altered in several types of cancers (15). Increased level of CYR61 mRNA in primary breast cancers is associated with more advanced features and poor prognosis (40). Moreover, the higher expression of CYR61 in tumor tissues predicts a higher cancer-specific mortality and a shorter survival duration in colorectal cancer patients (41). Higher expression of CYR61 is positively associated with short overall survival time in osteosarcoma patients (42). Circulatory CYR61 is positively associated with 3-month mortality in patients with chronic kidney diseases (42). Therefore, the correlations between the level of serum CYR61 on admission and the prognostic outcomes were estimated in CAP patients. We found that the number of patients with mechanical ventilation, vasoactive agent, ICU admission, death, and longer hospital stays was gradually elevated in parallel with the increased level of serum CYR61 among CAP patients. Moreover, the predictive capacities of serum CYR61 for severity and death were evaluated through ROC curve in CAP patients. These results indicated that the predictive powers of serum CYR61 for severity and death were higher compared with frequent indicators of inflammation and infection, such as CRP, IL-6, PCT, and D-dimer in CAP patients. Moreover, single serum CYR61 did not elevate the predictive capacities for severity and death compared with CAP severity scores. However, serum CYR61 in combination with CAP severity scores obviously upregulated the predictive powers for severity and death among CAP patients. Therefore, these results hinted that serum CYR61 on admission is positively associated with the adverse prognosis in CAP patients.

Interestingly, age may affect the relationships between serum CYR16 and CAP severity scores. The influence of serum CYR61 elevation on severity scores was stronger in CAP patients aged less than 62 years compared with those aged more than 62 years. In addition, serum CYR61 was increased in CAP patients with higher age compared with lower age. Our previous studies found that elderly COVID-19 patients are more vulnerable to suffering from multiple organ injury and adverse prognosis (43–45). Moreover, a previous study has demonstrated that older CAP patients have severer symptoms and longer hospital stays (46). Our data also indicated that the older patients have higher severity scores compared with patients aged less than 62.0 years. The possible reason is that older patients with CAP have worser resistance for disease. Therefore, older age partially aggravated the CAP severity and elevated the risks of poor prognosis among CAP patients.

CYR61, a member of the matrix secreted protein, is implicated in DNA virus and microbial infection establishment and virulence (47, 48). Virus infection can directly activate *Cyr61* promoter dependent on c-Jun N-terminal kinase (JNK) signal pathway and upregulate the expression of CYR61 in infectious cells (47). Lysophosphatidic acid (LPA) is a bioactive lysophospholipid that can bind to specific G protein-coupled receptors. Several studies have revealed that bacterial infection upregulates CYR61 mRNA levels through activated LPA receptors in epithelial cells (49). Moreover, TNF-α and IL-17 exposure can upregulate CYR61 level in different cells (50, 51). Nuclear factor kappa-B (NF-κB) and activator protein-1 (AP-1), two significant nuclear transcription factors, can regulate downstream inflammatory cytokines and chemokines (52). CYR61 is shown to activate NF-κB signaling pathway in macrophages and evokes the secretion of proinflammatory cytokines and chemokines (53). Besides, CYR61 directly upregulates the inflammatory cytokines production through binding of AP-1 complexes to inflammatory cytokines promoter (54). On the contrary, CYR61 inhibition can alleviate inflammation in mice (55). So, we believe that there is a positive feedback loop between inflammatory cytokines and CYR61. CYR61 elevation further upregulates the expression of inflammatory cytokines and chemokines in pulmonary epithelial cells, which finally evokes lung injury. As we all know, CAP is not only an inflammatory disease but also an infectious disease. Maybe, these are the possible reasons of CYR61 elevation in CAP patients. Consequently, CYR61 elevation may exert an important role in CAP progression.

Although this research has increased our awareness about CYR61 in CAP, it has some limitations. First, this topic was a single-center research, and more samples from multicenter research are needed in the future. Second, the expression of CYR61 was only detected in the serum. However, the levels of CYR61 were unknown in pulmonary tissues and alveolar lavage fluid of CAP patients. Third, this study had not clarified the underlying mechanism of CYR61 elevation. Additional animal and cellular experiments are needed to explore the exact mechanism of CYR61 increase in CAP patients. Fourth, serum CYR61 is not the unique indicator for CAP. The level of serum CYR61 on admission can evaluate the severity and poor prognosis. But, serum CYR61 can’t distinguish CAP or other inflammatory diseases. Finally, we are not sure whether CYR61 is the therapeutic target for CAP. Maybe more experiments *in vitro* and *in vivo* can resolve this question in the future.

## Conclusion

This study mainly estimated the associations of serum CYR61 with severity and prognosis in CAP patients through a prospective cohort study. Our results found that serum CYR61 is gradually elevated in parallel with severity scores in CAP patients. Serum CYR61 on admission is positively associated with severity and poor prognosis in CAP patients, and the positive dose-response associations are obvious. Serum CYR61 in combination with severity scores elevated the predictive capacities for severity and death compared with single serum CYR61 or CAP severity scores. Our evidence suggests that CYR61 is involved in the pathophysiology of CAP. Therefore, serum CYR61 may be used as a biomarker for the diagnosis and prognosis in CAP patients.

## Data availability statement

The raw data supporting the conclusions of this article will be made available by the authors, without undue reservation.

## Ethics statement

The studies involving human participants were reviewed and approved by the Ethics Committee of Second Affiliated Hospital of Anhui Medical University (YX2021-147). The patients/participants provided their written informed consent to participate in this study. Written informed consent was obtained from the individual(s) for the publication of any potentially identifiable images or data included in this article.

## Author contributions

LF and HZ conceived the study, contributed to its design and conception, and drafted the manuscript. M-XY, J-YC, YL, JS, D-XH, Q-YH, and H-YL contributed to study design and carried out the studies. All authors read and approved the final manuscript.

## Footnotes

1
https://www.cusabio.com/

2
http://www.jymbio.com/

## References

[1] Franco J. Community-acquired pneumonia. *Radiol Technol.* (2017) 88:621–36. 28900048

[2] Nair GB Niederman MS. Updates on community acquired pneumonia management in the ICU. *Pharmacol Ther.* (2021) 217:107663. 10.1016/j.pharmthera.2020.107663 32805298 PMC7428725

[3] Torres A Sibila O Ferrer M Polverino E Menendez R Mensa J Effect of corticosteroids on treatment failure among hospitalized patients with severe community-acquired pneumonia and high inflammatory response: a randomized clinical trial. *JAMA.* (2015) 313:677–86. 10.1001/jama.2015.88 25688779

[4] Ho PL Cheng VC Chu CM. Antibiotic resistance in community-acquired pneumonia caused by *Streptococcus pneumoniae*, methicillin-resistant *Staphylococcus aureus*, and *Acinetobacter baumannii*. *Chest.* (2009) 136:1119–27. 10.1378/chest.09-0285 19809053

[5] Rudan I O’Brien KL Nair H Liu L Theodoratou E Qazi S Epidemiology and etiology of childhood pneumonia in 2010: estimates of incidence, severe morbidity, mortality, underlying risk factors and causative pathogens for 192 countries. *J Glob Health.* (2013) 3:010401. 10.7189/jogh.03.010401 23826505 PMC3700032

[6] Cilloniz C Martin-Loeches I Garcia-Vidal C San Jose A Torres A. Microbial etiology of pneumonia: epidemiology, diagnosis and resistance patterns. *Int J Mol Sci.* (2016) 17:2120. 10.3390/ijms17122120 27999274 PMC5187920

[7] Cunha BA. Atypical pneumonias: current clinical concepts focusing on Legionnaires’ disease. *Curr Opin Pulm Med.* (2008) 14:183–94. 10.1097/MCP.0b013e3282f79678 18427241

[8] Self WH Wunderink RG Williams DJ Zhu Y Anderson EJ Balk RA *Staphylococcus aureus* community-acquired pneumonia: prevalence, clinical characteristics, and outcomes. *Clin Infect Dis.* (2016) 63:300–9. 10.1093/cid/ciw300 27161775 PMC4946021

[9] Vardakas KZ Matthaiou DK Falagas ME. Incidence, characteristics and outcomes of patients with severe community acquired-MRSA pneumonia. *Eur Respir J.* (2009) 34:1148–58. 10.1183/09031936.00041009 19541719

[10] Nair H Nokes DJ Gessner BD Dherani M Madhi SA Singleton RJ Global burden of acute lower respiratory infections due to respiratory syncytial virus in young children: a systematic review and meta-analysis. *Lancet.* (2010) 375:1545–55. 10.1016/S0140-6736(10)60206-1 20399493 PMC2864404

[11] Shi T Balsells E Wastnedge E Singleton R Rasmussen ZA Zar HJ Risk factors for respiratory syncytial virus associated with acute lower respiratory infection in children under five years: systematic review and meta-analysis. *J Glob Health.* (2015) 5:020416. 10.7189/jogh.05.020416 26682048 PMC4676580

[12] Brigstock DR. The CCN family: a new stimulus package. *J Endocrinol.* (2003) 178:169–75. 10.1677/joe.0.1780169 12904165

[13] Lau LF. CCN1/CYR61: the very model of a modern matricellular protein. *Cell Mol Life Sci.* (2011) 68:3149–63. 10.1007/s00018-011-0778-3 21805345 PMC3651699

[14] Grzeszkiewicz TM Kirschling DJ Chen N Lau LF. CYR61 stimulates human skin fibroblast migration through Integrin alpha vbeta 5 and enhances mitogenesis through integrin alpha vbeta 3, independent of its carboxyl-terminal domain. *J Biol Chem.* (2001) 276:21943–50. 10.1074/jbc.M100978200 11287419

[15] Terada N Kulkarni P Getzenberg RH. Cyr61 is a potential prognostic marker for prostate cancer. *Asian J Androl.* (2012) 14:405–8. 10.1038/aja.2011.149 22343491 PMC3472413

[16] Moon HG Kim SH Gao J Quan T Qin Z Osorio JC CCN1 secretion and cleavage regulate the lung epithelial cell functions after cigarette smoke. *Am J Physiol Lung Cell Mol Physiol.* (2014) 307:L326–37. 10.1152/ajplung.00102.2014 24973403 PMC4137167

[17] Perkowski S Sun J Singhal S Santiago J Leikauf GD Albelda SM. Gene expression profiling of the early pulmonary response to hyperoxia in mice. *Am J Respir Cell Mol Biol.* (2003) 28:682–96. 10.1165/rcmb.4692 12760966

[18] Shimada I Matsui K Brinkmann B Hohoff C Hiraga K Tabuchi Y Novel transcript profiling of diffuse alveolar damage induced by hyperoxia exposure in mice: normalization by glyceraldehyde 3-phosphate dehydrogenase. *Int J Legal Med.* (2008) 122:373–83. 10.1007/s00414-008-0226-6 18301909

[19] Kurundkar AR Kurundkar D Rangarajan S Locy ML Zhou Y Liu RM The matricellular protein CCN1 enhances TGF-β1/SMAD3-dependent profibrotic signaling in fibroblasts and contributes to fibrogenic responses to lung injury. *FASEB J.* (2016) 30:2135–50. 10.1096/fj.201500173 26884454 PMC4871800

[20] Du Y Ding Y Chen X Mei Z Ding H Wu Y MicroRNA-181c inhibits cigarette smoke-induced chronic obstructive pulmonary disease by regulating CCN1 expression. *Respir Res.* (2017) 18:155. 10.1186/s12931-017-0639-1 28806967 PMC5557525

[21] Tan ZX Fu L Wang WJ Zhan P Zhao H Wang H Serum CYR61 Is associated with airway inflammation and is a potential biomarker for severity in chronic obstructive pulmonary disease. *Front Med.* (2021) 8:781596. 10.3389/fmed.2021.781596 34917638 PMC8669148

[22] Kase Y Okano H. Expression of ACE2 and a viral virulence-regulating factor CCN family member 1 in human iPSC-derived neural cells: implications for COVID-19-related CNS disorders. *Inflamm Regen.* (2020) 40:32. 10.1186/s41232-020-00143-6 32934757 PMC7485212

[23] Lee SJ Zhang M Hu K Lin L Zhang D Jin Y. CCN1 suppresses pulmonary vascular smooth muscle contraction in response to hypoxia. *Pulm Circ.* (2015) 5:716–22. 10.1086/683812 26697179 PMC4671746

[24] Woo YJ Seo Y Kim JJ Kim JW Park Y Yoon JS. Serum CYR61 is associated with disease activity in graves’ orbitopathy. *Ocul Immunol Inflamm.* (2018) 26:1094–100. 10.1080/09273948.2017.1319960 28548552

[25] Samarpita S Ganesan R Rasool M. Cyanidin prevents the hyperproliferative potential of fibroblast-like synoviocytes and disease progression via targeting IL-17A cytokine signalling in rheumatoid arthritis. *Toxicol Appl Pharmacol.* (2020) 391:114917. 10.1016/j.taap.2020.114917 32044269

[26] Feng CM Cheng JY Xu Z Liu HY Xu DX Fu L Associations of serum resistin with the severity and prognosis in patients with community-acquired pneumonia. *Front Immunol.* (2021) 12:703515. 10.3389/fimmu.2021.703515 34858392 PMC8630736

[27] Wang JL Chen X Xu Y Chen YX Wang J Liu YL The associations of serum IL-37 with the severity and prognosis in patients with community-acquired pneumonia: a retrospective cohort study. *Front Immunol.* (2021) 12:636896. 10.3389/fimmu.2021.636896 34025645 PMC8138168

[28] Fei J Fu L Cao W Hu B Zhao H Li JB. Low vitamin D status is associated with epithelial-mesenchymal transition in patients with chronic obstructive pulmonary disease. *J Immunol.* (2019) 203:1428–35. 10.4049/jimmunol.1900229 31427443

[29] Fu L Fei J Tan ZX Chen YH Hu B Xiang HX Low vitamin D status is associated with inflammation in patients with chronic obstructive pulmonary disease. *J Immunol.* (2021) 206:515–23. 10.4049/jimmunol.2000964 33361208 PMC7812059

[30] Feng CM Wang XM Li MD Xu Z Hua DX Cheng JY Serum interleukin-17 predicts severity and prognosis in patients with community acquired pneumonia: a prospective cohort study. *BMC Pulm Med.* (2021) 21:393. 10.1186/s12890-021-01770-6 34856971 PMC8637026

[31] Zheng L Fei J Feng CM Xu Z Fu L Zhao H. Serum 8-iso-PGF2α predicts the severity and prognosis in patients with community-acquired pneumonia: a retrospective cohort study. *Front Med.* (2021) 8:633442. 10.3389/fmed.2021.633442 33869248 PMC8044775

[32] Cao LF Cheng JY Xu Z Feng CM Zhao H Wang XM Serum 8-Hydroxydeoxyguanosine is a potential indicator for the severity and prognosis in patients with community-acquired pneumonia: a prospective cohort study. *J Immunol.* (2022) 208:321–7. 10.4049/jimmunol.2100711 34911771

[33] Jiang X Huang CM Feng CM Xu Z Fu L Wang XM. Associations of Serum S100A12 with severity and prognosis in patients with community-acquired pneumonia: a prospective cohort study. *Front Immunol.* (2021) 12:714026. 10.3389/fimmu.2021.714026 34745092 PMC8569229

[34] Fu L Zhao H Xiang Y Xiang HX Hu B Tan ZX Reactive oxygen species-evoked endoplasmic reticulum stress mediates 1-nitropyrene-induced epithelial-mesenchymal transition and pulmonary fibrosis. *Environ Pollut.* (2021) 283:117134. 10.1016/j.envpol.2021.117134 33866216

[35] Zheng L Jiang YL Fei J Cao P Zhang C Xie GF Circulatory cadmium positively correlates with epithelial-mesenchymal transition in patients with chronic obstructive pulmonary disease. *Ecotoxicol Environ Saf.* (2021) 215:112164. 10.1016/j.ecoenv.2021.112164 33756289

[36] Jiang YL Fei J Cao P Zhang C Tang MM Cheng JY Serum cadmium positively correlates with inflammatory cytokines in patients with chronic obstructive pulmonary disease. *Environ Toxicol.* (2022) 37:151–60. 10.1002/tox.23386 34652871

[37] Qin HY Li MD Xie GF Cao W Xu DX Zhao H Associations among S100a4, sphingosine-1-phosphate, and pulmonary function in patients with chronic obstructive pulmonary disease. *Oxid Med Cell Longev.* (2022) 2022:6041471. 10.1155/2022/6041471 35165531 PMC8837900

[38] Fu L Chen YH Xu S Yu Z Zhang ZH Zhang C Oral cholecalciferol supplementation alleviates lipopolysaccharide-induced preterm delivery partially through regulating placental steroid hormones and prostaglandins in mice. *Int Immunopharmacol.* (2019) 69:235–44. 10.1016/j.intimp.2019.01.052 30738993

[39] Liu HY Xiang HX Xiang Y Xu Z Feng CM Fei J The associations of serum S100A9 with the severity and prognosis in patients with community-acquired pneumonia: a prospective cohort study. *BMC Infect Dis.* (2021) 21:327. 10.1186/s12879-021-06020-y 33827454 PMC8028176

[40] Xie D Nakachi K Wang H Elashoff R Koeffler HP. Elevated levels of connective tissue growth factor, WISP-1, and CYR61 in primary breast cancers associated with more advanced features. *Cancer Res.* (2001) 61:8917–23. 11751417

[41] Jeong D Heo S Sung Ahn T Lee S Park S Kim H Cyr61 expression is associated with prognosis in patients with colorectal cancer. *BMC Cancer.* (2014) 14:164. 10.1186/1471-2407-14-164 24606730 PMC3975645

[42] Liu Y Zhang F Zhang Z Wang D Cui B Zeng F High expression levels of Cyr61 and VEGF are associated with poor prognosis in osteosarcoma. *Pathol Res Pract.* (2017) 213:895–9. 10.1016/j.prp.2017.06.004 28647210

[43] Fu L Li XY Fei J Xiang Y Xiang HX Li MD Myocardial injury at early stage and its association with the risk of death in COVID-19 patients: a hospital-based retrospective cohort study. *Front Cardiovasc Med.* (2020) 7:590688. 10.3389/fcvm.2020.590688 33195480 PMC7661636

[44] Fu L Fei J Xu S Xiang HX Xiang Y Hu B Liver dysfunction and its association with the risk of death in COVID-19 patients: a prospective cohort study. *J Clin Transl Hepatol.* (2020) 8:246–54. 10.14218/JCTH.2020.00043 33083246 PMC7562804

[45] Xiang HX Fei J Xiang Y Xu Z Zheng L Li XY Renal dysfunction and prognosis of COVID-19 patients: a hospital-based retrospective cohort study. *BMC Infect Dis.* (2021) 21:158. 10.1186/s12879-021-05861-x 33557785 PMC7868870

[46] Fung HB Monteagudo-Chu MO. Community-acquired pneumonia in the elderly. *Am J Geriatr Pharmacother.* (2010) 8:47–62. 10.1016/j.amjopharm.2010.01.003 20226392

[47] Kim SM Park JH Chung SK Kim JY Hwang HY Chung KC Coxsackievirus B3 infection induces cyr61 activation via JNK to mediate cell death. *J Virol.* (2004) 78:13479–88. 10.1128/JVI.78.24.13479-13488.2004 15564459 PMC533934

[48] Kurozumi K Hardcastle J Thakur R Shroll J Nowicki M Otsuki A Oncolytic HSV-1 infection of tumors induces angiogenesis and upregulates CYR61. *Mol Ther.* (2008) 16:1382–91. 10.1038/mt.2008.112 18545226 PMC2659780

[49] Emre Y Imhof BA. Matricellular protein CCN1/CYR61: a new player in inflammation and leukocyte trafficking. *Semin Immunopathol.* (2014) 36:253–9. 10.1007/s00281-014-0420-1 24638890

[50] Lin SK Kok SH Lee YL Hou KL Lin YT Chen MH Simvastatin as a novel strategy to alleviate periapical lesions. *J Endod.* (2009) 35:657–62. 10.1016/j.joen.2009.02.004 19410078

[51] Zhang Q Wu J Cao Q Xiao L Wang L He D A critical role of Cyr61 in interleukin-17-dependent proliferation of fibroblast-like synoviocytes in rheumatoid arthritis. *Arthritis Rheum.* (2009) 60:3602–12. 10.1002/art.24999 19950293

[52] Cao J Xu T Zhou C Wang S Jiang B Wu K NR4A1 knockdown confers hepatoprotection against ischaemia-reperfusion injury by suppressing TGFβ1 via inhibition of CYR61/NF-κB in mouse hepatocytes. *J Cell Mol Med.* (2021) 25:5099–112. 10.1111/jcmm.16493 33942481 PMC8178266

[53] Bai T Chen CC Lau LF. Matricellular protein CCN1 activates a proinflammatory genetic program in murine macrophages. *J Immunol.* (2010) 184:3223–32. 10.4049/jimmunol.0902792 20164416 PMC2832719

[54] Li H Li H Huo R Wu P Shen Z Xu H Cyr61/CCN1 induces CCL20 production by keratinocyte via activating p38 and JNK/AP-1 pathway in psoriasis. *J Dermatol Sci.* (2017) 88:46–56. 10.1016/j.jdermsci.2017.05.018 28602508

[55] Zhou M Ze K Hua L Liu L Kuai L Zhang M Cyr61 promotes inflammation of a gouty arthritis model in rats. *Mediators Inflamm.* (2020) 2020:8298615. 10.1155/2020/8298615 32774151 PMC7396108

